# The Role of Working Memory in the Processing of Scalar Implicatures of Patients With Schizophrenia Spectrum and Other Psychotic Disorders

**DOI:** 10.3389/fpsyg.2021.635724

**Published:** 2021-05-06

**Authors:** Walter Schaeken, Linde Van de Weyer, Marc De Hert, Martien Wampers

**Affiliations:** ^1^Brain and Cognition, Faculty of Psychology and Educational Sciences, KU Leuven, Leuven, Belgium; ^2^University Psychiatric Center KU Leuven, Leuven, Belgium; ^3^Center for Clinical Psychiatry, Department of Neurosciences Antwerp Health Law and Ethics Chair, University of Antwerp, Antwerp, Belgium

**Keywords:** pragmatics, experimental pragmatics, scalar implicature, schizophrenia spectrum and other psychotic disorders, working memory

## Abstract

A number of studies have demonstrated pragmatic language difficulties in people with Schizophrenia Spectrum and Other Psychotic Disorders. However, research about how people with schizophrenia spectrum and other psychotic disorders understand scalar implicatures (SIs) is surprisingly rare, since SIs have generated much of the most recent literature. Scalar implicatures are pragmatic inferences, based on linguistic expressions like *some*, *must*, *or*, which are part of a scale of informativeness (e.g., *some/many/all*). Logically, the less informative expressions imply the more informative ones, but pragmatically people usually infer that the presence of a less informative term implies that the more informative term was not applicable. In one of the few existing studies with people with schizophrenia spectrum and other psychotic disorders, Wampers et al. (2018) observed that in general, people with schizophrenia spectrum and other psychotic disorders were less likely to derive SIs than controls. The current study has three main aims. First, we want to replicate the original finding with the scalar terms *some-all*. Second, we want to investigate how these patients deal with different scalar terms, that is, we want to investigate if scalar diversity is also observed in this clinical group. Third, we investigate the role of working memory, often seen as another important mechanism to enable inferring SIs. Twenty-one individuals with a psychotic disorder and 21 matched controls answered 54 under-informative statements, in which seven different pairs of scalar terms were used. In addition, working memory capacity was measured. Patients with schizophrenia spectrum and other psychotic disorders did not make more logical interpretations when processing quantifiers, disconfirming Wampers et al. (2018). However, certain scalar scales elicited more pragmatic interpretations than others, which is in line with the scalar diversity hypothesis. Additionally, we observed only partial evidence for the role of working memory. Only for the scalar scale *and-or*, a significant effect of working memory was observed. The implications of these results for patients with schizophrenia spectrum and other psychotic disorders are discussed, but also the role of working memory for pragmatic inferences, as well as the place of SIs in experimental pragmatics.

## Introduction

Pragmatics is the study of the use of language in context, whereby one of the key findings is that the meaning of words and sentences can change in the light of the specific context they are used. Important in this domain is the distinction between the literal and the intended meaning (in Grice, 1975). The distinction between literal and intended meaning can be experimentally studied using for instance metaphors, humor, or irony (for a recent overview, see Noveck, 2018). Other linguistic expressions which are widely researched in experimental pragmatics are Scalar Implicatures (SIs). Expressions like *some-many-all may-should-must*, *warm-hot* are part of a scale organized by informativity (Horn, 1972). These scales have a specific characteristic: when the stronger term holds, the weaker term also holds, while the opposite is not true. Consider a professor correcting essays who tells her partner in the evening: “*Some of the essays were thought-provoking*.” This expression is true when the professor found all essays thought-provoking and when she did find only some of them thought-provoking but not all. However, in case the professor only found some and not all of the essays thought-provoking, she could not say “*All of the essays were thought-provoking*” if she did not want to lie. One can notice here already the Gricean distinction (1975) between what is said on the one hand (the specific sentence) and what is implicated on the other hand (the speaker’s meaning). An implicature is a component of the speaker’s meaning, which is not said and therefore should be inferred. How do listeners make the required inference in our first example according to Grice? First of all, listeners adhere to the Cooperation principle and assume that a speaker is trying to be cooperative. More specifically, and translating this to the Gricean maxim of Quantity, a listener expects that the speaker was as informative as possible and also that she gave as much information as was needed (and also not more information than needed). Since our professor used the weaker term of a scale (e.g., *some essays were thought-provoking*) and not the stronger term (e.g., *all essays were thought-provoking*), the listener can infer that the professor found that the stronger term was not appropriate, otherwise she would have used it. Consequently, the listener can infer that, or stated differently, the listener enriches the original expression to the upper-bounded meaning with “*some and not all of the essays were thought-provoking*.” Noveck (2001) refers to this line of reasoning as the preference for the pragmatic interpretation above the logical one. Experimental research, often only focusing on *all-some*, clearly demonstrates that adults predominantly prefer the pragmatic upper-bounded *some but not all* interpretation of *some* (e.g., Noveck, 2001; Bott and Noveck, 2004; De Neys and Schaeken, 2007; Marty and Chemla, 2013; Heyman and Schaeken, 2015; van Tiel and Schaeken, 2017). Children, however, prefer more often than adults the logical meaning of *some* which is also compatible with *all* (see e.g., Chierchia et al., 2001; Noveck, 2001; Papafragou and Musolino, 2003; Foppolo et al., 2012; Janssens et al., 2014, 2015; Schaeken et al., 2019), although more adult-like behavior can be elicited (e.g., Papafragou and Musolino, 2003; Pouscoulous et al., 2007; Barner et al., 2011; Katsos and Bishop, 2011; Schaeken et al., 2019).

It is clear that there is abundant experimental research on SIs, and it is sometimes said that *some-all* expressions stand as the poster child of pragmatic inference (Scontras et al., 2018). There are several reasons for the special status of these expressions in experimental pragmatics: the context and content of such expressions are simple to manipulate; potential confounding variables are easy to control; competing theories make clear predictions about these experimental manipulations; different age-groups be tested with similar paradigms (see e.g., Noveck and Sperber, 2007; Katsos and Cummins, 2010). Therefore, it is surprising that few researchers have addressed the issue how clinical populations deal with SIs.

The current study wants to fill this gap in knowledge, linking understanding SIs by people with schizophrenia spectrum and other psychotic disorders with scalar diversity and the role of working memory. The remaining part of this introduction is structured in the following way. First, we will describe pragmatic difficulties of people with schizophrenia spectrum and other psychotic disorders. Next, we will discuss some findings with respect to SIs with clinical populations, more specifically people with Autism Spectrum Disorder (ASD). Then, we will discuss briefly the potential role of Theory of Mind (ToM) and more extensively the role of working memory. Finally, we will introduce the issue of scalar diversity and describe our own research.

According to the American Psychiatric Manual (APA 5) Schizophrenia Spectrum and Other Psychotic Disorders is characterized by for instance delusions, hallucinations, but also by disorganized speech and other symptoms that cause social or occupational dysfunction. Language impairments have always been observed in people with schizophrenia spectrum and other psychotic disorders. Extensive research showed that patients diagnosed with schizophrenia spectrum and other psychotic disorders have difficulties when they have to deal with non-literal expressions or the non-literal parts of expressions. Studies focusing on one or a few aspects, like humor, irony, metaphors, proverbs, … showed that these aspects are all difficult to understand for patients with schizophrenia spectrum and other psychotic disorders (see e.g., Langdon et al., 2002a,b; Sponheim et al., 2003; Brüne and Bodenstein, 2005; Linscott, 2005; Bambini et al., 2016, 2020; for an overview, see e.g., Bosco and Parola, 2017). Also, broad assessments of the pragmatic competence showed a deficit in patients with schizophrenia spectrum and other psychotic disorders. Colle et al. (2013) found evidence of a wide dysfunction using the Assessment Battery of Communication; Bambini et al. (2016) showed, using the APACS Test for the assessment of pragmatic abilities and cognitive substrates that the pragmatic abilities were weakened in schizophrenia, with comprehending discourse and non-literal meanings being especially compromised. Bambini et al. (2016) even argue that the high frequency of impairment suggests that the pragmatic deficit is a core feature of schizophrenia. The latter study also evidenced the role of pragmatics for quality of life: overall pragmatic qualities predicted quality of life, while this was not the case for other cognitive variables in their study. In the same line, Adamczyk et al. (2016) showed that selective language and communication skills (inferential meaning, humor and metaphors, emotional and linguistic prosody) are important for patients with schizophrenia in their social recovery process. Agostoni et al. (2021) show through a mediation analysis that pragmatics has both a direct and an indirect effect on daily functioning, and especially in interpersonal functioning. In other words, recent evidence not only points to pragmatic difficulties in people with schizophrenia spectrum and other psychotic disorders, but also to the important role pragmatics might play for their daily functioning.

As said before, SIs is one of the most widely researched topics in pragmatic with adults and typically developing children. However, research on clinical populations is unexpectedly scarce, with as main exception research about the understanding of SIs by people with ASD. In many of these studies no decrease in pragmatic responses was observed (see e.g., Pijnacker et al., 2009; Chevallier et al., 2010; Su and Su, 2015; Hochstein et al., 2017; see also Antoniou et al., 2016 and Heyman and Schaeken, 2015 for similar findings with participants with higher Autism-Spectrum Quotient scores). However, some other studies did observe differences, albeit sometimes subtle (see e.g., Nieuwland et al., 2010; Zhao et al., 2015; Mazzaggio and Surion, 2018; Schaeken et al., 2018).

Regarding people with schizophrenia spectrum and other psychotic disorders, there is to the best of our knowledge only one published study on the understanding of SIs. Wampers et al. (2018) observed in both a binary and ternary statement-evaluation-task with *some-all* that patients with schizophrenia spectrum and other psychotic disorders derived fewer SIs than matched control participants. Moreover, ToM abilities were positively correlated with deriving SIs.

This significant correlation between ToM abilities and the derivation of SIs in Wampers et al. (2018) added to the mixed evidence on the role of ToM for pragmatics. ToM skills are often seen as an important driver of pragmatic comprehension. Support for this claim comes from work with typically developing adults, for instance showing an important involvement of ToM skills in irony (e.g., Andrés-Roqueta and Katsos, 2017). Even more evidence come from work with clinical populations, like ASD and schizophrenia spectrum and other psychotic disorders, where a relation between the ToM deficit and their difficulty in pragmatics is observed (see e.g., Baron-Cohen, 1988; Happé, 1993; Corcoran et al., 1995, 1997; Langdon et al., 2002a; Janssen et al., 2003; Brüne and Bodenstein, 2005), or from work where impaired pragmatic reasoning is observed in patients with cortical lesions to ToM areas (Champagne-Lavau and Joanette, 2009; Spotorno et al., 2015). However, this picture is far from consistent. Some authors (e.g., Langdon et al., 2002a; Mazza et al., 2008) observed for instance a role of ToM for irony, but not for metaphors, while others (e.g., Brüne and Bodenstein, 2005; Mo et al., 2008) observed the opposite pattern. Similarly, sometimes effects were already observed for first-order ToM (see Happé, 1993), sometimes only for second-order ToM (see Mo et al., 2008; Panzeri and Foppolo, 2016). Finally, the picture of ToM for schizophrenia spectrum and other psychotic disorders is even more complicated, since ToM is not impaired in paranoid schizophrenia, which is often characterized by a hyper-ToM (Abu-Akel and Bailey, 2000; Frith, 2004; Peyroux et al., 2019).

Similarly, the role of ToM for scalars is debatable. Pijnacker et al. (2009) argue that the ToM load for scalars is low: A SI seems to require only first order mental states (e.g., *she knows, or she does not know that*…) and not second-order mental states (e.g., *she does not know that he knows that*…). Therefore, it is possible that just basic ToM skills are already sufficient for inferring SIs (see also Chevallier et al., 2010). Brüne (2003) argues that the comprehension of this first order ToM is relatively preserved in patients with schizophrenia spectrum and other psychotic disorders. Moreover, Andrés-Roqueta and Katsos (2017) argue that especially in work with children the knowledge needed to infer the SIs is often visually accessible, therefore minimizing the demand on ToM.

One possibility mentioned by Wampers et al. (2018) to explain their observed ToM-effect, is the potential role of working memory. Both ToM and working memory are considered as cognitive substrates underlying pragmatic competence (see e.g., Cummings, 2017). However, they are not independent of each other, since for instance working memory capacity is required to be able to think about other persons thoughts. The role of working memory for pragmatic language understanding is widely discussed. For instance, Chiappe and Chiappe (2007) and Columbus et al. (2015) showed the important role of working memory in metaphor comprehension for young adults. Bambini et al. (2021) observed that working memory skills were crucial for the pragmatic skills tested (comprehension of oral narrative stories, humor, figurative language and implicatures). Flexibility played a role for figurative language and implicatures, while, surprisingly, inhibition was not a robust predictor. Also for clinical populations and definitely also for patients with Schizophrenia Spectrum and Other Psychotic Disorders, an important role of working memory is observed (see Forbes et al., 2009), also in pragmatics. For instance, Bosia et al. (2016) observed a significant correlation of working memory with pragmatic production, Kiang et al. (2007) with comprehension of proverbs and Schettino et al. (2010) with idiomatic expressions. Moreover, the role of working memory in the production of SIs is a popular research topic and a vast amount of experiments evidenced a processing cost associated with processing SIs. When given less time, participants infer less SIs (see e.g., Bott and Noveck, 2004; van Tiel and Schaeken, 2017); similarly, when working memory was burdened, pragmatic inferences dropped (see e.g., De Neys and Schaeken, 2007; Huang and Snedeker, 2009; Dieussaert et al., 2011; Marty et al., 2013). For this reason, we decided to focus solely on working memory in this study. However, to be fair, not all evidence points in the same direction. Grodner et al. (2010) observed no delay for pragmatic inferences from *some* compared to other, non-scalar expressions and in the latent class analysis of Heyman and Schaeken (2015), working memory capacity did not explain the interindividual variability in the interpretation of infelicitous *some* statements (see e.g., also Feeney et al., 2004; Breheny et al., 2013; Janssens and Schaeken, 2016).

Past research almost uniquely focused on *or-and*, *might-must* and especially *some-all*, whereby it was basically assumed that other scales would behave similarly. However, recent research (see e.g., Doran et al., 2009; Geurts, 2010; Simons and Warren, 2018) questioned this uniformity. In a series of experiments, Van Tiel et al. (2016) showed that different types of scales are not all the same and we cannot use one type as the prototypical type. They tested 43 types of scalar inferences by presenting participants a statement with the weaker scalar term (e.g., *or*), and asking them if they would infer that the corresponding sentence with the stronger scalar term (e.g., *and*) is false. The results showed large differences across different lexical scales. Almost none of the participants made this falsity-inference with pairs as *content-happy* or *tired-exhausted*, while almost all of them made it for pairs like *possible-certain*, and *some-all*. As potentially relevant factors for the scalar diversity closed versus open scales, minimal versus rich contexts, word class and semantic distance are mentioned (see also Gotzner et al., 2018).

## Experiment

The current experiment aims first of all to replicate the observed difficulty of patients with schizophrenia spectrum and other psychotic disorders with SIs with quantifiers, since that was to the best of our knowledge the first observation of it (Wampers et al., 2018). We opted for the use of a more fine-grained scale with a middle option as in the second experiment of Wampers et al. (2018) (see also Katsos and Bishop, 2011; Schaeken et al., 2018) instead of a task with the classic binary answer options. We hypothesize to observe similar effects with respect to the quantifier items, in other words, we expect the patients with schizophrenia spectrum and other psychotic disorders to interpret the quantifiers less pragmatically than the control group. Moreover, we want to investigate if these patients demonstrate scalar diversity as well or whether their difficulty is more uniform. In previous studies (e.g., Van Tiel et al., 2016), typically developing adults produce especially for quantifiers, disjunctions and modals a higher number of pragmatic responses. In Wampers et al. (2018) the clinical group produced fewer pragmatic inferences on the quantifiers, although this significant decrease was not large. Combining these two evidences, we expect our clinical group to produce fewer pragmatic responses for the quantifiers, disjunctions and modals than the control group, but still to a higher degree than for the other items, for which typically developing adults predominantly produce logical responses. Finally, we want to investigate if working memory capacity is related to the number of pragmatic responses given. It is well-documented that the working memory capacity of patients with schizophrenia spectrum and other psychotic disorders is decreased (see e.g., Goldman-Rakic, 1994; Silver, 2003; Lee and Park, 2005; Forbes et al., 2009; Arnsten, 2013; Nielsen et al., 2017). Therefore, it makes it very interesting to investigate for the first time the working memory and scalar diversity observations with this group. We hypothesize that working memory capacity will definitely influence the items on which the control group produces a higher number of pragmatic responses (i.e., quantifiers, disjunctions and modals). In order to obtain these aims, we presented seven different scales to our participants, which consisted of a group of patients with schizophrenia spectrum and other psychotic disorders and a control group, while we also tested their working memory capacity.

### Method

#### Participants

In total, 42 persons participated in the experiment (22 men and 20 women). Half of these participants (11 men and 10 women) with a mean age of 27.5 (SD = 4.99) were diagnosed with Schizophrenia Spectrum and Other Psychotic Disorders according to DSM-V criteria by an experienced psychiatrist. All patients were hospitalized at the moment of testing. The second half of the participants, the control group, was matched to the patient group with respect to age and educational level (see Table 1) and consisted of 21 adults (11 men, 10 women) with a mean age of 27.0 (SD = 5.42). All participants were of Dutch literacy and provided written informed consent. The study was granted full ethical approval by the Ethics Committee of the University Psychiatric Hospital KU Leuven.

**TABLE 1:** Demographic variables of patient and control group.

	***N***	**Age**	**Gender**		**Education**			
			**Men**	**Women**	**EE**	**SE**	**HE**	**UE**
Patient group	21	27.5	11	10	3	12	2	4
Control group	21	27.0	11	10	0	14	3	4
Total	42	27.2	22	20	3	26	5	8

*EE is elementary school education only, SE is secondary school education, HE is higher education, and UE is university education.*

#### Material

For the assessment of the sensitivity to SIs, we constructed a questionnaire taken from the Dutch items of Van Tiel et al. (2016) and Zevakhina (2012). The questionnaire contained 54 under-informative sentences subdivided into 35 critical items and 19 filler items. To exclude sequence effects, four randomized versions of the questionnaire were prepared. For each item, a fictional person named Vera made a statement that contained a scalar term and could give rise to a scalar implication. Next, the participants were asked whether it could be deduced that, according to Vera, the statement implied that a stronger scalar term was not involved. The assessment was made by means of a five-point Likert scale, ranging from 1 = totally disagree to 5 = completely agree. An example of a critical item is:

*Vera says: “Some theater performances are interesting.”*

*Would you infer from this that, according to Vera, not all theater performances are interesting?*

The 35 critical items were subdivided into seven pairs of different scalars, whereby each pair had five critical items: existential quantifier items (*all-some, always-sometimes*), disjunctive items (*and-or*), modal items (*have to-may*), and four pairs of adjective items (*excellent-good*, *hot-warm*, *huge-big*, *terrible-bad*). The critical items can be found in Appendix 1. The questionnaire also contained 19 filler items, of which 13 were valid and six were invalid. The valid and invalid filler items are also listed in Appendix 1. These items are superficially similar to the critical items, but they are pragmatically or semantically clearly wrong. With these items, we could therefore also test whether or not our participants had sufficient language and reasoning capacities. An example of a valid control item is:

*Vera says: “The garden is small.”*

*Would you deduce from this that, according to Vera, the garden is not large?*

An example of an invalid control item:

*Vera says: “The sea is warm.”*

*Would you deduce from this that, according to Vera, the sea is not clear blue?*

The 52 statements of each stimulus set were bundled in random order in booklets that displayed one item per page to discourage participants to return to previous responses. The first page of each booklet contained the task instructions. On the last page participants filled in their age, gender and educational level.

Working memory was assessed by means of the Digit Span subtest (with three parts, that is listen to sequences of numbers orally and to repeat them (a) as heard, (b) in reverse order, and (c) in ascending order) of the Dutch version of the fourth edition of the Wechsler Adult Intelligence Scale (WAIS IV). The subtests’ scores were converted on the basis of the test manual into a standardized working memory score.

#### Procedure

Each participant voluntarily participated in the study and signed the information and consent form. The participants were tested individually in a quiet room. The experiment took approximately 30 min per participant. The measurement of the working memory capacity was taken together with the researcher and lasted about 15 min. It took approximately 15 min to complete the implicature questionnaire.

#### Statistical Analysis

Overall performance on the filler items was good (86% for the control group, 82% for the clinical group). In line with Van Tiel et al. (2016); see also (Pipijn, 2014), participants who answered less than 14 out of 19 of the filler items correctly were excluded from the analyses. This implied that 4 of the control subjects and 5 of the participants with schizophrenia spectrum and other psychotic disorders were excluded from the analyses. Even after the exclusion of these participants, both groups did not differ significantly in terms of age and educational level.

The average performance of the included participants on the filler items was 92% for the control group and 89% for the clinical group.

The responses obtained on the five-point Likert scale were transposed into a tertiary score (1 and 2 were collapsed into “disagree,” 3 was “neutral,” and 4 and 5 were collapsed into “agree”). Given the ordinal character of tertiary scores, we performed a mixed effect ordinal regression analysis with the tertiary agreement score as the dependent variable. The independent variables were Group (with the levels schizophrenia spectrum and other psychotic disorders group and matched control group), Scalar-Type (with the seven different types of scalar terms) and Working Memory Capacity as measured by the standardized Digit Span score. The latter score was mean-centered. The model was fitted using the clmm() function from the ordinal package in R (Christensen, 2015). All models included random intercepts for participants and items and a random slope for scalar type to capture the extent to which the possible mean differences between scalar types may differ across participants.

We started with the most complex fixed effects structure including the three-way interaction between group, scalar type and working memory capacity besides all two-way interactions and main effects. Subsequently we used backward elimination which involved simplifying the model by removing interaction terms that did not contribute significantly as evaluated through a likelihood ratio test. We verified the final model fitting by evaluating whether Akaike’s Information Criterion (AIC) would have led to the same conclusion. In all analyses we used an alpha level of 0.05.

### Results

Table 2 presents the percentage of answers in each response category for each scalar type as a function of participant group and working memory capacity. Low and high working memory capacity are defined as a standardized DS score below or above the population mean, respectively.

**TABLE 2:** Percentages of each response type for each scalar type as a function of group and working memory capacity.

		**Control group**		**Clinical group**	
**Scalar type**	**Response options**	**Low WM**	**High WM**	**Low WM**	**High WM**
Disjunctions	Disagree	60.00	86.67	40.00	75.00
	Neutral	12.00	6.67	0.00	12.50
	Agree	28.00	6.67	60.00	12.50
Good–excellent	Disagree	48.00	48.33	42.50	30.00
	Neutral	16.00	21.67	30.00	40.00
	Agree	36.00	30.00	27.50	30.00
Big-huge	Disagree	44.00	50.00	57.50	35.00
	Neutral	16.00	11.67	20.00	32.50
	Agree	40.00	38.33	22.50	32.50
Modal	Disagree	60.00	65.00	45.00	47.50
	Neutral	12.00	10.00	12.50	20.00
	Agree	28.00	25.00	42.50	32.50
Quantifier	Disagree	96.00	90.00	87.50	70.00
	Neutral	0.00	10.00	2.50	15.00
	Agree	4.00	0.00	10.00	15.00
Bad-horrible	Disagree	44.00	48.33	57.500	37.50
	Neutral	16.00	13.33	10.00	22.50
	Agree	40.00	38.33	32.50	40.00
Warm-hot	Disagree	36.00	53.33	47.50	45.00
	Neutral	32.00	10.00	22.50	22.50
	Agree	32.00	36.67	30.00	32.50

The final model included one two-way interactions, that is, the interaction between Scalar-Type and Working Memory Capacity. For a complete description of the final model, see Table 3.

**TABLE 3:** Complete description of the final model*.

**Random effects**									
**Groups**	**Name**	**Variance**	**Std.Dev.**	**Correlations**					
Participant	(intercept)	3.62	1.91						
Item	(intercept)	0.37	0.61						
	Big-Huge	1.27	1.13	0.18					
	Modal	3.01	1.74	−0.75	0.36				
	Quantifier	8.42	2.90	0.30	0.54	0.34			
	Bad-horrible	1.31	1.14	−0.14	0.93	0.67	0.56		
	Warm-hot	1.37	1.17	−0.88	0.28	0.89	−0.09	0.57	
	Disjunction	7.33	2.71	−0.63	−0.06	0.57	−0.32	0.21	0.70
**Number of groups**	**Participant**	**Item**							
	33	35							
**Fixed effects**									
Coefficients	Estimate	Std.Error	*z*-value	Pr(>| z|)					
Psychosis	0.3217	0.4537	0.709	0.4783					
ST “Big-huge”	−0.1636	0.5060	−0.323	0.7465					
ST “Modal”	−0.4735	0.5560	−0.852	0.3944					
ST “Quantifier”	−4.9910	1.1917	−4.188	0.0000281					
ST “Bad-horrible”	0.0290	0.5043	0.058	0.9541					
ST “Warm-hot”	−0.0650	0.4976	−0.131	0.8961					
ST “Disjunction”	−1.5558	0.7184	−2.166	0.0303					
DS score	−0.0669	0.1278	−0.524	0.6005					
DS score × ST “Big-huge”	0.0371	0.1063	0.349	0.7271					
DS score × ST “Modal”	−0.0827	0.1318	−0.627	0.5304					
DS score × ST “Quantifier”	−0.0170	0.2498	−0.068	0.9457					
DS score × ST “Bad-horrible”	0.0541	0.1062	0.507	0.6118					
DS score × ST “Warm-hot”	0.0751	0.1045	0.718	0.4725					
DS score × ST “Disjunction”	−0.4597	0.1941	−2.369	0.0178					

*The fixed effects are: Group [with levels: Psychosis and age and education matched control subjects (=reference group)], ST = Scalar Type [with levels good-excellent (=reference group), big-huge, modals, quantifiers, bad-horrible, warm-hot, disjunctions], DS score = scaled Digit Span score from the WAIS, mean-centered so that the intercept reflects the results of a participant with the mean Digit Span score in the total study population.

As can be seen in Table 3 two scalar types, that is, quantifiers (*Z* = −4.18, *p* < 0.000) and disjunctions (*Z* = −1.56, *p* = 0.0303) differed significantly from the scalar type that acted as the reference category i.e., “Good-Excellent.” Additional pairwise comparisons using emmeans() showed that quantifiers are also interpreted more pragmatically than the adjective items “Big-Huge” (*Z* = −4.143, *p* = 0.0007), “Warm-Hot” (*Z* = −4.048, *p* = 0.0010), “Bad-Horrible” (*Z* = −4.324, *p* = 0.0003), and the modal items (*Z* = −3.808, *p* = 0.0027). No significant interpretative differences were observed between quantifier and disjunctive items (*Z* = −2.513, *p* = 0.1546).

Although the results in Table 3 show that disjunctive items were interpreted significantly more pragmatically than items from the reference scalar type (Good- excellent), we observed no other significant pairwise differences were between disjunctions and other scalar types. This observation is probably due to the fact that the disjunctive scalar type is involved in a significant interaction with working memory capacity.

The significant interaction between the mean-centered measure of working memory capacity and the scalar type disjunctives (β = −0.46, *Z* = −2.37, *p* = 0.018) shows that the extent to which disjunctive items are interpreted pragmatically, depends on participants’ working memory capacity. Participants with a lower working memory capacity will interpret disjunctive items more logically than participants with a higher working memory capacity. The higher someone’s working memory capacity, the more he/she tends to interpret disjunctive items pragmatically. This can also be observed in Table 2.

**FIGURE 1:**
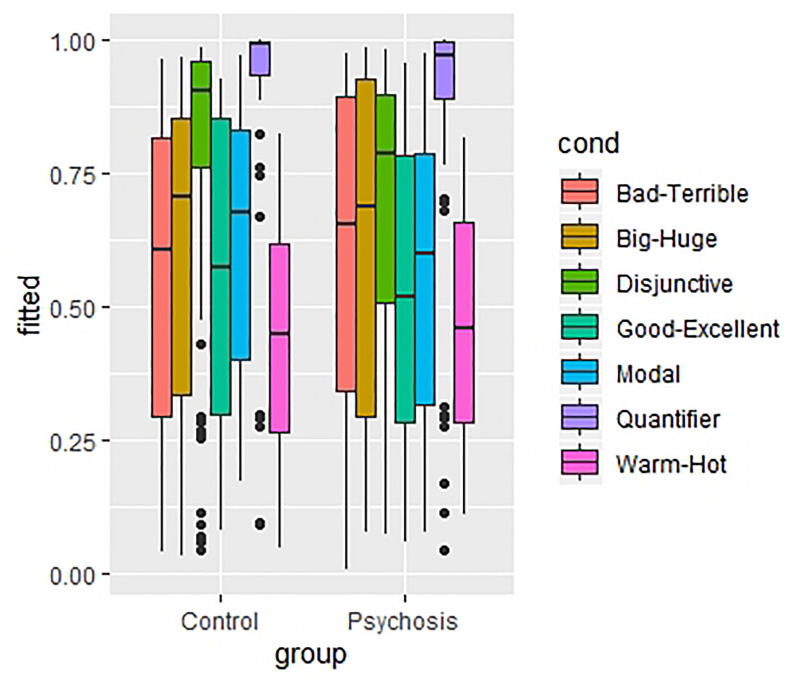
The boxplots of the fitted values of the final model for the different scalar types for control participants and participants diagnosed with Schizophrenia Spectrum and Other Psychotic Disorders.

There was no significant interaction between group and scalar type, so people diagnosed with Schizophrenia Spectrum and Other Psychotic Disorders and matched control subjects show the same response pattern when confronted with a diversity of scalar items. The similarity between both study populations is also illustrated in Figure 1 which shows the boxplots of the fitted values of the final model for the different scalar types for control subjects and subjects diagnosed with Schizophrenia Spectrum and Other Psychotic Disorders.

## General Discussion

As mentioned in the literature review, despite the abundant evidence that people with schizophrenia spectrum and other psychotic disorders show pragmatic difficulties, not much is known about how they deal with SIs. The first aim of the current study was to fill this gap in an attempt to replicate the only study about this topic (Wampers et al., 2018) in which fewer pragmatic responses were given by people with psychosis on SIs with quantifiers when compared with controls. As a second aim, the study broadened the SIs used and investigated if we can observe scalar diversity in people with schizophrenia spectrum and other psychotic disorders, as is shown in typically developed adults (see e.g., Van Tiel et al., 2016). As a third aim, the study investigated if working memory capacity is related to the number of pragmatic responses given. Our study found evidence for two of these three aims, but clearly in a nuanced way.

Starting with the results regarding the first aim, our study did not replicate the effect of Wampers et al. (2018): although our clinical group performed less pragmatically on the quantifier items than the control group, this effect was not significant. Hence, we could not confirm the hypothesis that the pragmatic difficulties of people with schizophrenia and other psychotic disorders can be extended to SIs. These results are therefore in contrast with Wampers et al. (2018), who did observe such a significant difference. Regarding the second aim, as hypothesized, we observed scalar diversity in our data: disjunctive and quantifier items were treated more pragmatically than the adjectives. These findings are in line with recent findings (see e.g., Van Tiel et al., 2016), which show that not all scalar items elicit a similar number of pragmatic responses. With respect to the third aim, an effect of working memory was observed, but again only in a subtle way: only the disjunctive items were solved less pragmatically by the people with lower working memory capacity.

How can we explain these results? We observed that especially disjunctive and quantifier items elicited more pragmatic responses and that the adjectives are answered more logically. This result ties well with previous studies, although we used a ternary scale instead of a binary scale. That a different procedure did lead to more or less the same results adds to the robustness of the scalar diversity effect. Interestingly, there was no significant effect of the clinical condition on scalar diversity. In other words, the clinical group showed more or less the same pattern of results as the control group. A potential explanation can be found in the study of Moro et al. (2015). They presented people with schizophrenia sentences in which they had to detect anomalies. Some of these sentences contained syntactic errors (violations of Universal Grammar principles) or semantic errors, resulting from a contradiction in the computation of the whole sentence meaning. The people with schizophrenia had only difficulties in identifying syntactic anomalies, suggesting an impairment of syntactic knowledge in schizophrenia. There were, however, no difficulties observed with semantic anomalies. The absence of difficulties with identifying semantic errors points to the absence of a semantic deficit. This could explain the lack of an effect of the clinical condition on scalar diversity, since the major hypotheses for scalar diversity mentioned in the literature are of a semantic nature (e.g., closed versus open scales, minimal versus rich contexts, word class, positively versus negatively oriented scalar words, and semantic distance) and hence will have a more or less equal influence on the control and the clinical group, as the absence of a main effect of group also shows. This observation is important with respect to the claim in the introduction of Bambini et al. (2016) that the high frequency of impairment suggests that the pragmatic deficit is a core feature of schizophrenia. The fact that there were no differences between our two groups with respect to scalar diversity runs against this idea, or at least, it nuances this thesis in showing that not all domains of pragmatic language are impaired. This important nuance is even more strengthened in the next point.

Contrary to our hypothesis and to Wampers et al. (2018), this study has been unable to demonstrate that for quantifiers there is a difference between the clinical and the control group. What might cause the differences between our results and those of Wampers et al. (2018) There are two important procedural differences between the current study and Wampers et al. (2018). The latter presented 10 quantifier items and only quantifier items, while in the current study only five quantifier items were presented, which were, moreover, mixed with many other scalar terms. Dieussaert et al. (2011) demonstrated that when participants have to change strategy often (in their case, by manipulating the number of filler items; in the current study by presenting seven different scalar items), the number of pragmatic responses decreases. It might therefore be that the performance of our control group was more logically compared to a study where only (and more) quantifiers were presented. Moreover, the tasks for the participants also clearly differed between the two studies. Wampers et al. (2018) used a statement-evaluation-task, where participants were presented with10 underinformative quantifier items like “*Some oaks are trees*” and they were asked to judge them as either true (logical) or false (pragmatic). This paradigm fits quantifiers very well, but it is incompatible with many other scalar expressions. Therefore, as in Pipijn (2014), Van Tiel et al. (2016), and Zevakhina (2012), the current study employs an inference paradigm, which, in general, leads to higher rates of SIs than the statement-evaluation paradigm (Geurts and Pouscoulous, 2009). In other words, it might be that these procedural differences caused the observed difference between the two studies on the quantifiers with respect to the effect of clinical group.

However, these conflicting data also accord with the mixed evidence with participants with ASD. On the one hand, some studies (e.g., Pijnacker et al., 2009; Chevallier et al., 2010; Su and Su, 2015) observed a similar amount of SIs with quantifiers for participants with ASD as for typically developing participants. Also, Hochstein et al. (2017) observed no difference in the amount of SIs between adolescents with ASD and controls. However, despite the ASD-group showing awareness of speakers’ mental states, they were not always considering these spontaneously when deriving SIs. On the other hand, there are two recent studies in which children with ASD answered less pragmatically on SIs than typically developing children (Schaeken et al., 2018; Mazzaggio et al., 2021).

Therefore, it is still under debate whether participants from clinical populations, and in this specific study participants with schizophrenia spectrum and other psychotic disorders, are less pragmatic with quantifiers than typically developing control participants. It is clear that more research is needed, not only with the current population, but also with other clinical populations. Therefore, it looks to us that SIs with quantifiers developed from the poster child of pragmatic inference (Scontras et al., 2018) into a capricious teenager. We still see the possibilities, the intrinsic promises of SIs with quantifiers as a key element in experimental pragmatics, but getting them realized is definitely challenging.

Our data regarding the role of working memory are partly in line with the literature (see the introduction for more details on the controversial nature of the role of working memory), finding a small and specific working memory effect, that is, on disjunctive items. Most relevant for our study presumably is van Tiel et al. (2019), which combined investigating scalar diversity with manipulations of working memory load with typically developed adults. Their study revealed an interesting significant interaction between memory load and scalar type. Greater memory load led to fewer pragmatic responses for four scales (*or-and*, *might-must*, *some-all*, and *most-all*), but for three scales there was no working memory effect at all (*low-empty*, *scarce-absent*, and *try-succeed*). Hence, like our study, these findings not only demonstrate scalar diversity, but also a nuanced working memory effect. However, contrary to our results, they did also find a working memory effect on the quantifiers and modals. Three important differences between their and our study might cause this difference: a statement-evaluation-task versus an inference paradigm, the answer options offered to the participants (a binary option versus 5 options) and, definitely important, the fact that we only measured working memory and therefore treated it as a interindividual difference variable, while in van Tiel et al. (2019) working memory load was a manipulated factor (see also Dieussaert et al., 2011 for a discussion of measuring and manipulating working memory). What makes disjunctions special so that in both studies the inference from “*or*” to “*not and*” is cognitively costly? There are different potential accounts, but an intriguing explanation can be found in Singh et al. (2016). They argue that the retrieval of alternatives for disjunctions is peculiar, since there are two mechanisms for generating alternatives for adults (lexical replacement and the possibility of deleting material to generate an alternative), while there is only one for children (lexical replacement), giving rise to the different number of pragmatic responses by children and adults (see also Tieu et al., 2017; Verschueren et al., 2004). It might be that this developmental difference is also linked to working memory capacity, in which more working memory capacity is needed for the two roads to the alternatives. Future research should clarify this possible link.

Given the role of pragmatics for quality of life, intervention studies are critical tools in the rehabilitation process. Recently, some promising intervention or remediation studies have been developed for clinical and older populations (see e.g., Tompkins et al., 2011; Blake et al., 2013; Gabbatore et al., 2015, Gabbatore et al., 2017; Lundgren and Brownell, 2016; Bambini et al., 2020; Parola et al., 2020). For example, the PragmaCom (Bambini et al., 2020) focuses on the use of the Gricean maxims to strengthen the appreciation and knowledge of the pragmatic processes in communication, and uses for instance metaphors, proverbs, humor, and off-topic verbosity. The outcome of our study with respect to SIs suggests that adding them to such training programs, albeit interesting, is not essential. The outcome with respect to disjunctions, however, suggest that adding a working memory component in intervention studies could strengthen them (see Cortese et al., 2014; Danielsson et al., 2015; Spencer-Smith and Klingberg, 2015).

Before concluding, we have to mention some limitations of our study. First, a working memory manipulation would be a stronger indicator of a potential working memory effect than the measurement that we used in the current study. Second, the diagnosis of our rather young group of patients was general. It would be interesting if future research could investigate an older group of patients, and definitely with more specific information about the diagnosis. This seems especially relevant given the cognitive heterogeneity of people with schizophrenia spectrum and other psychotic disorders (see e.g., Van Rheenen et al., 2017a,b; Buonocore et al., 2021). Related is the absence of direct IQ and language measurements. In our experiment, we matched our participant on educational level, since it is associated with many life outcomes and functions, such as income, occupation, intelligence, and language. There is indeed abundant evidence that education is a significant driver of language proficiency (see e.g., Massing and Schneider, 2017; Rudd and Honkiss, 2020). Therefore, we used educational level as a proxy for language proficiency. Moreover, the filler items used are also an implicit test of basic language and reasoning abilities. Average accuracy of the total group of participants on those items was good (86% for the control group, 82% for the clinical group). Moreover, to be sure of the basic language and reasoning abilities of our participants, those who scored less than 14 out of 19 were excluded, which lead to the exclusion of 9 participants (4 in the control group and 5 in the clinical group). The average accuracy on the filler items for the included participants was 92 and 89%, respectively, clearly indicating good and comparable language and reasoning skills of the participants in our sample. However, given the important role of language proficiency (see e.g., Parola et al., 2020) and verbal IQ (e.g., Chevallier et al., 2010) for pragmatic understanding, a more direct measurement would have been better and is definitely a recommendation for future studies. Third, it would be interesting to add additional measurements apart from working memory. There is not so much work on executive functions and implicatures with typically developing adults and the evidence is mixed. Antoniou et al. (2016) observed that working memory predicts the amount of pragmatic scalar responses, but inhibition did not. Fairchild (2018) reported significant correlations between executive functions and pragmatic, but when factoring ToM, these correlations disappeared. Husband (2014), however, did observe an effect of executive function. In other domains of pragmatics, and especially with clinical populations, executive functions played an important role. Bosia et al. (2016) for instance observed significant correlations between processing of figurative language and verbal memory, while humor was correlated with verbal memory, verbal fluency and processing speed in patients with schizophrenia. With respect to proverbs interpretations by patients with schizophrenia, the role of executive functions is clearly determined: set shifting and planning in Sponheim et al. (2003), divided attention, set-shifting and inhibitory control in Thoma et al. (2009) and cognitive flexibility in Mossaheb et al. (2014). Especially inhibitory control seems to be important in clinical studies (see e.g., Li et al., 2017; Parola et al., 2020). Bambini et al. (2021) found that in the elderly inhibition was not a significant predictor, but cognitive flexibility played a significant role in pragmatic comprehension in the elderly. Hence, future studies could fruitfully explore this issue further with SIs by including executive functions like inhibition, set shifting and cognitive flexibility. Furthermore, Brüne and Bodenstein (2005) and Champagne-Lavau and Stip (2010), investigating cognitive and executive functions and ToM together, both observed that ToM seems to be a better predictor than the cognitive and executive functions. Hence, future research should ideally not use only a measurement of different executive functions but also of ToM.

## Conclusion

Overall, our study adds new knowledge, both theoretically and clinically, to the field of clinical and experimental pragmatics. From a theoretical point of view, the most obvious implication of the current study is the importance of taking into account scalar diversity, not only when working with typically developed adults, but also with clinical groups: one cannot generalize from *some* scalar expressions. Moreover, the role of working memory has been confirmed, but, importantly, only in a nuanced way, that is, we only observed a reliable effect for the disjunction items. From a clinical point of view, this study does not support the finding of Wampers et al. (2018) that people with schizophrenia spectrum and other psychotic disorders have difficulties with the pragmatic interpretation of quantifiers. However, our study accords with the hypothesis that there is no severe semantic deficit in our clinical group, given the observed scalar diversity effect. Finally, given the link between pragmatic functioning and quality of life, the current results might be used to feed intervention studies. Our study seems to underline the potential role of working memory training in intervention programs for people with schizophrenia spectrum and other psychotic disorders.

## Data Availability Statement

The raw data supporting the conclusions of this article will be made available by the authors, without undue reservation.

## Ethics Statement

The studies involving human participants were reviewed and approved by Ethics Committee of the University Psychiatric Center KU Leuven. The patients/participants provided their written informed consent to participate in this study.

## Author Contributions

WS and MW did the data interpretation, wrote the general discussion and designed the study, with the help of MD. LV prepared the experiment, constructed the stimuli, and performed the experiment. MW did the statistical analysis. WS wrote the first draft of the introduction and method section. All authors approved the final version of the manuscript.

## Conflict of Interest

The authors declare that the research was conducted in the absence of any commercial or financial relationships that could be construed as a potential conflict of interest.
